# Institutionalising ELSA in the moment of breakdown?

**DOI:** 10.1186/2195-7819-10-1

**Published:** 2014-01-03

**Authors:** Ellen-Marie Forsberg

**Affiliations:** Oslo and Akershus University College, P.O. Box 4, St. Olavs plass, 0130 Oslo, Norway

**Keywords:** ELSA research, Responsible research and innovation (RRI), Institutionalism, Legitimacy

## Abstract

This article discusses outcomes of a dialogue conference on ‘The road ahead for ELSA in Norway: Issues of quality, influence and network cooperation’ held in Oslo in December 2012. Norwegian researchers in the field of ethical, legal and social aspects of technologies (ELSA) were invited to discuss conceptual and strategic issues, as well as the setup of a researcher network. In the article I take an institutionalist approach and discuss challenges in institutionalising an ELSA network at a time when a designated ELSA funding programme is coming to an end. The research question is how the Norwegian ELSA network can succeed as a persistent network in times of greater uncertainties. The article claims that the network needs to gain legitimacy, outlines different dimensions of legitimacy and interprets the conference discussions in light of these dimensions. Central challenges and success factors facing the ELSA network are discussed and the article concludes with reflections on the potential future of ELSA in Norway. Although the article has a Norwegian context, the discussions in the article are likely to be relevant for researchers all across Europe, as similar developments are taking place also elsewhere in the European research funding context.

## Introduction

In December 2012 the Ethical, Legal and Social Aspects of biotechnology, nanotechnology and neurotechnology (ELSA) programme of the Research Council of Norway (RCN) and the Research Group on Responsible Innovation at the Oslo and Akershus University College (HiOA) jointly organised a dialogue conference ‘The road ahead for ELSA in Norway: Issues of quality, influence and network cooperation’. Norwegian researchers in the field of ethical, legal and social aspects of technologies (ELSA) were invited to discuss conceptual and strategic issues, as well as the setup of a research network (ELSA Norway). This research network was to be researcher initiated and to receive two years of funding from the ELSA programme.

A dialogue conference is traditionally a tool used for organisational development where there is a need for a common discussion of status, need for changes and possible solutions and strategies for further development (see Pålshaugen 1998). The method is based upon work life democracy and empowerment of employees. Traditionally, it tries to temporarily neutralise hierarchy based power by carrying out most discussions in smaller (heterogeneous) groups. The idea is that agreement on specific actions can be achieved even if there is not a shared understanding of the situation. In addition to the democratic justification for this approach, it will ideally give room for otherwise marginalised expertise and may lead to strategies that are more widely anchored in the organisation.

The ELSA conference was not a traditional dialogue conference in the sense that the ELSA community of researchers^a^ and the RCN ELSA programme are not institutionally one organisation. However, the researchers and the programme need each other as part of a research system (assumed to be) to the benefit of society. Moreover, the dialogue conference had more plenary lectures than usual for this method. This was motivated by the insight that the discussions are not unique to Norway: such conceptual and strategic issues are discussed by many scholars and communities in and outside Europe (for reference, please see the EMBO Science & Society Series on Convergence Research^b^, including articles from some of the conference key note speakers). Four international key note speakers were invited to give their perspectives on ELSA, integrated research and the current development of responsible research and innovation (RRI). In addition, representatives of the Research Council presented information on the ELSA programme as well as on ELSA dimensions of closely bordering programmes (BIOTEK2021 and NANO2021)^c^.

The conference programme was set up by a small steering group consisting of representatives of some central Norwegian ELSA communities, in dialogue with representatives from the RCN ELSA programme. There were two group work sessions. In the first the participants could choose between the following group topics:

- **Group 1:** Theoretical ELSA, integrated projects and RRI: In what directions should Norway go?
  - What are the conceptual and strategic implications of the different alternatives?
- **Group 2:** Where and when should the ELSA research make a difference?
  - What is implied in integrating ELSA in other programs? What are the implications of the different approaches for the quality of ELSA research?
- **Group 3:** What is ELSA research and who are ELSA researchers?
  - How to conceptualise our core competencies and value across our internal disciplinary differences? What kinds of competencies are needed in the future?
- **Group 4:** Optional: Other topics (at the conference this was defined as ‘Law, political theory and economics’)

In the second group session all the groups (preset by the organisers to ensure a good mix of participants in the groups) discussed the practicalities of the planned Norwegian ELSA network:

- What should be the objective of the network?
- Who should be involved/enrolled/mobilised? Why? And what should they contribute with?
- What do we want from the website?
- What activities should the network prioritise? Examples: seminars, Ph.D courses, anthologies, international activities, newsletters, input to policy, societal dialogue?

The groups were asked to consider the first two years as well as the longer term.

Minutes were taken in the plenary sessions and these were distributed to the participants in a conference report.

In this article I wish to discuss in a theoretical perspective one of the main topics of the ELSA conference, namely the question of how the Norwegian ELSA network can succeed as a persistent network in times of greater uncertainty with regard to funding. In order to answer this research question I will apply an institutionalist approach, focusing especially on Suchman’s theory of legitimacy (Suchman 1995). Although such a theoretical framework was not applied in the dialogue conference I will show how the conference discussions illustrate the different legitimacy dimensions identified from the literature. This amounts to a validation and enrichment of the theoretical analysis. The quotations cited in this article are from the conference report. The quotes should not be interpreted as representative of the whole ELSA community, of the participants at the dialogue conference in general, or even of all in the group stating them. The points expressed in the plenary as outcomes of the group work were sometimes simply references to individual opinions voiced in the group.

Based on the analyses, I will recommend some general strategies for the ELSA network in Norway. Though this paper has a Norwegian focus I believe that the analyses in the paper also have relevance for similar conceptual-strategic constructs in other countries. Ruth Chadwick spoke at the conference about similar challenges for the ESRC Genomics Network (EGN) in the UK. Moreover, in a European context, similar challenges have been worrying established ELSA communities (whether they call themselves ELSA, applied bioethics, constructive technology assessment or similar terms) in the transition from the European Commission 7^th^ framework programme to the new research funding programme Horizon 2020. In general, the legitimacy dimensions discussed in this article are claimed by Suchman (for instance Suchman 1995) to be generic for all organisations (individual companies, public authorities, networks, etc.) and the discussions in this paper should therefore be relevant for a range of organisations that are faced with existential threats. In the field of research this article should be particularly relevant for all research communities that are facing new requirements of an existential nature, such as new expectations of interdisciplinarity, user orientation and increased societal relevance.

In this paper I will first briefly describe the institutional perspective to be applied, then I will use a simple institutionalist framework to analyse the outcomes of the conference and to discuss challenges and success factors for the network. I will conclude the paper with some reflections on the value of having such a network.

As an organiser and participant of the dialogue conference, and as a participant in the ELSA Norway Network I necessarily occupy an insider position with regard to the topics discussed in the article. However, I believe the impartiality of the analyses in the article is not affected by this position, and that my experience from the field instead strengthens the analyses.

## A description of the institutional perspective to be applied

The perspective offered in this article is that of institutionalism. I will argue that the conference can be seen as an initiative to institutionalise a new organisation. In this perspective, the ELSA conference was effectively the start of the institutionalisation of the ELSA network. This network is to be institutionalised at the same time that the most important funding source of the network is to be terminated. The second ELSA programme period is concluded in 2014, and by the time of the conference it was clear that the Research Council would not continue the programme into a third period. It was communicated in the conference that the ELSA programme had helped the ELSA researchers get started, and now we had to organise ourselves to continue on our own. Although an organisation could simply be set up without any conference the idea behind the conference was that it should contribute to consolidating the community by focusing on shared concerns and strategies. In this paper I will therefore interpret the conference discussions in terms of institutional theory. But first I will briefly review the parts of institutional theory that I will apply in this article.

Institutional theory, developed in the 1970s, emphasises the dependence of modern organisations on their environments (Meyer 2008, p. 790). Institutional theory is considered relevant in many fields, including governance (Cashore 2002 Forsberg 2012a), change management (Henisz and Zelner 2003), applied ethics (Boyle et al. 2001 Forsberg et al. 2012) and networks and strategic alliances (Dacin et al. 2007). In my account here I draw on an institutionalist framework that I have found useful also in an analysis of a regional innovation network (Forsberg 2012b), although the challenges discussed are somewhat different.

Within institutional theory there are different approaches. For the purpose of this paper (with a focus on institutionalising a network), Scott’s distinction between understanding organisations as *rational*, *natural* and *open systems* is useful (Scott 1987). According to Boyle et al., the *rational* systems approach understands the organisation as having ‘(1) a visible set of hierarchical authority relations in which (2) work activities are governed by formal rules and clearly defined criteria for evaluation, relations that (3) are designed to pursue some set of goals.’ (2001: 31). When an organisation is analysed as a *natural* system, the focus is on the informal aspects of the organisation, acknowledging that individuals may have a significant influence on attitudes and conduct in an organisation and that cultures and sub-cultures may flourish more or less decoupled from formal structures, often undermining, replacing or transforming them (ibid., p. 33). The open systems approach deals with the relation between the organisation and its environment, on which it is ‘dependent for resources, personnel, and legitimacy’ (ibid. 2001, p. 35). As the main challenge currently faced by the ELSA community revolves around changes in the environment affecting the availability of resources the open systems approach is the relevant perspective to apply in this paper. In this paper I will therefore take the institutionalist notion of organisations as open systems as an analytic starting point, and will demonstrate how the issue of legitimacy can be applied in order to understand challenges and success factors in establishing an interdisciplinary network such as the ELSA network.

Legitimacy has traditionally been discussed in the context of governments, but since the 1970s the concept has also been applied to organisations, inter-organisational relations and actions. In his seminal paper, Suchman (1995) describes organisational legitimacy as an anchor point for understanding organisation-environment conditions (p. 571) and claims that ‘[l]egitimacy is a generalized perception or assumption that the actions of an entity are desirable, proper, or appropriate within some socially constructed system of norms, values, beliefs, and definitions’ (p. 574). Suchman has a rich review and discussion of several aspects of legitimacy. We do not need to consider all the details of his model, but will focus on two dimensions particularly relevant for the legitimacy of networks. The first dimension deals with pragmatic, moral and cognitive legitimacy. *Pragmatic legitimacy* ‘rests on the self-interested calculations of an organization’s most immediate audiences’ (p. 578). *Moral legitimacy* ‘reflects a positive normative evaluation of the organization and its activities’ (p. 579). *Cognitive legitimacy* may involve a taken-for-grantedness of the organisation, or at least its comprehensibility, that is, that the audiences understand the place of the organisation and its activities (p. 582). Suchman also distinguishes between *gaining*, *maintaining* or *repairing* legitimacy, and suggests that different legitimating strategies are relevant in these different situations.

Human and Provan (2000) claim to be the first contribution applying legitimacy theory to networks (p. 359). They have been followed by Kumar and Das (2007) on interpartner legitimacy in alliance development processes and Person et al. (2011) on interpartner legitimacy in regional strategic networks. Low and Johnston (2010) have analysed legitimacy related to firms’ access to strategic networks. Forsberg (2012b) analysed dynamics in regional innovation networks related to dimensions of legitimacy. The purpose of this article is not to further institutional theory, but rather to use it as a tool for better understanding the challenges facing the ELSA community. In the article I will apply central dimensions of Suchman’s legitimacy theory and will relate relevant parts of the discussions in the dialogue conference to these dimensions. I will discuss different strategies the network may choose in order to gain legitimacy among targeted audiences and through this survive as a dynamic research network over time.

### The ELSA network as a new institution

I will here treat the ELSA network as a new institution that needs to gain legitimacy. One may of course deny that the ELSA network amounts to a new institution, suggesting that the ELSA community has been institutionalised through the ELSA programme since the programme’s start. However, there is reason to doubt this. A definition of an institution is a ‘more-or-less taken-for-granted repetitive social behavior that is underpinned by normative systems and cognitive understandings that give meaning to social exchange and thus enable self-reproducing social order. Institutions are characterized by lack of overt enforcement, their survival resting upon “relatively self-activating social processes” (Greenwood et al. 2008, pp. 4-5). Put in simpler terms, an institution can be characterised as a social unit with a common understanding of ‘this is the way we do it here’. So even if there indeed was an ELSA community (which consisted of the individuals that were invited to the dialogue conference), I will claim that it was not institutionalised. There was no rational organisation, and it is hard to see that there was a common culture throughout the community, as will be better spelled out below.

According to several participants in the programme, the two periods of the ELSA programme (ELSA I 2002-2007 and ELSA II 2008-2014) have been characterised by a high degree of controversy on the design of the programme. There have been disagreements about the relative importance of applied ethics and science and technology studies (STS) in the programme board and calls for proposals. And there have been disagreements about the appropriate balance between empirical and theoretical projects, and between disciplinary, interdisciplinary and transdisciplinary projects. In particular, there has been discussion about the setup of so-called integrated projects, where social science and humanist researchers cooperate in projects with natural scientists and technologists, and about the extent to which such projects should be prioritised in the programme. It is therefore hard to claim that there have been many taken-for-granted practices or an established social order that has defined the ELSA research community.

Except for the common funding source, and the fact that all the projects combined humanist, legal or social science perspectives to technology related topics, it is hard to point to substantial commonalities in approaches and interests among those that can be characterised as Norwegian ELSA researchers. It is also hard to define the borders of the ELSA community. In the dialogue conference the list of invited participants was a result of the steering committee’s own knowledge of the community, combined with a list from the Research Council of project leaders of projects funded in the ELSA programme. I will therefore claim that the ELSA community had not been institutionalised at the point of the dialogue conference. With the ELSA network, the institutionalisation of the community would be strengthened by establishing a rational organisation, with a structure and goals. In addition to such a rational organisation, however, there is a need to establish some sort of informal organisation; i.e. a common culture or identity that would function as “glue” in the organisation. The dialogue conference amounted to an important step in building such a common cultural basis. However, the dialogue conference was not an attempt to streamline the community into a common research approach, rather to explore differences and similarities. An important focus was on what the community shared; in terms of concerns, problem understandings and wishes for the future. What do we have in common that could function as a cultural basis for institutionalising the network?

## ELSA Norway and the need to gain legitimacy

The inconvenient truth forming the background for the dialogue conference was that the main source of existence for the community, the RCN ELSA programme, was coming to an end. In an institutionalist perspective the most important external agent for the community has been the RCN ELSA programme and this agent has had a defining function for the ELSA community. With the end of the ELSA programme, the relation between the ELSA community and the Research Council is changing and the worst case scenario is that these changes will lead to a breakdown in Norwegian ELSA. From having a designated programme sympathetic to the internal worries of the community, the community will in the future have to deal with programmes that have other main interests. ELSA related projects will from 2014 received funding from the Research Council either through the so-called ‘large programmes’, BIOTEK2021 or NANO2021 programmes, or through research programmes related to humanistic or social science research. These are broader programmes where ELSA research is likely to play a less central role. European research programmes are also likely to play a larger role in the funding of Norwegian ELSA research. The community is painfully aware of this change and the new needs for the community to become a stronger agent towards this emerging environment.

However, the ELSA community does not start from scratch. ELSA research has legitimacy also in parts of the Research Council other than the ELSA programme. Over more than 10 years ELSA research has been funded by the biotechnology and nanotechnology programmes and currently both the national biotechnology and nanotechnology strategies emphasise the importance of ELSA aspects in these fields. From the earlier functional genomics (FUGE) and nano materials (NANOMAT) programmes to the current BIOTEK2021 and NANO2021 programmes ELSA research has been included. NANO2021 has a budget of NOK 92 million yearly and BIOTEK2021 has approximately NOK 213 million yearly^d^. 2 to 5% of the funding of these programmes is allocated to ELSA research. As ELSA inherently is related to technologies, and historically especially to bio-and nanotechnology, proper integration of ELSA in the BIOTEK2021 and NANO2021 programmes is important. If ELSA research fails to be acknowledged in these programmes the identity of ELSA–as opposed to other humanistic or social science research that is not inherently related to emerging technologies–is threatened. The ELSA network therefore needs strong legitimacy in these research and innovation programmes.

Inclusion of ELSA in technology programmes has generally been organised as calling for natural science/technology projects with an ELSA component. However, over time there has been increasing emphasis on so-called ‘integrated projects’. Integrated projects are inspired by work of scholars such as David Guston, on integrating ELSA researchers into practical technology development work; Erik Fisher, on midstream modulation or socio-technical integration in which this integration is focused in laboratories on facilitating reflexive practices amongst the scientists; as well as Arie Rip, on constructive technology assessment (see for instance Guston and Sarewitz 2002 Fisher et al. 2012 and Schot and Rip 1997). Such approaches are responses to the criticism of earlier ELSA research for being too isolated and simply an ‘add-on’ to the natural science research. In both strategies for including ELSA research in technology programmes ELSA researchers need to reach out to the natural scientists and the nano and biotech industry in order to be included in their technology development (or other natural science) projects. The ELSA researchers need to appear as a legitimate partner to these environments.

From the ELSA researchers’ perspective there are two challenges with funding ELSA research over large scale innovation programmes. There is a risk that ELSA research may simply be regarded as an addition to the ‘real’ (i.e. natural science and technology) research and thus be defined only in reference to the innovation (or other natural science) research. This will make it harder for the ELSA researchers to set the premises for the research. A second, and related, risk is that the RCN nanotechnology and biotechnology programme boards and officers will not necessarily see the need for building up ELSA research in itself, but rather use ELSA research simply as instrumental to innovation projects, perhaps not taking into account that the capacity and quality of ELSA research need to be safeguarded if it is to function in the best–instrumental or non-instrumental–way. Therefore my claim is that ELSA research in the context of these programmes needs to gain legitimacy as strands of research with equal integrity as the natural sciences.

Similar challenges are likely to be connected with ELSA related projects in Horizon 2020. In European Commission research funding responsible research and innovation (RRI) is gaining increased importance as a cross-cutting approach embedding ELSA in larger innovation projects. RRI is a conceptual invention, launched in a specifically European innovation policy context that states that researchers and funding agencies need to consider the wider societal implications of the innovations that are triggered by their work. This responsibility cannot merely be implemented in the context of ‘applications’; it needs to be integrated during the process of agenda-setting. The approach assumes that there can be no successful implementation of techno-scientific knowledge without a proper embedding in society. Von Schomberg (2012, p. 50) defines RRI in the following way: ‘Responsible research and innovation is a transparent, interactive process by which societal actors and innovators become mutually responsive to each other with a view on the (ethical) acceptability, sustainability and societal desirability of the innovation process and its marketable products (in order to allow a proper embedding of scientific and technological advances in our society)’. (von Schomberg 2012, p. 50). Other definitions are given by for instance Owen, Macnaghten and Stilgoe (2012) (see also the contribution from Zwart et al. in this themed issue).

There are also other Norwegian programmes, like the RCN SAMKUL programme on cultural assumptions of societal development (including technological developments), that may fund Norwegian ELSA related research. The SAMKUL programme is a social science and humanities based programme with a budget of NOK 350 million for the period of 2011-2013. ELSA related research is one out of seven themes in SAMKUL. A large part of ELSA researchers have humanistic or social science backgrounds and naturally belong in this program, and it therefore does not seem as important to legitimise a network vis-à-vis SAMKUL as towards the technology oriented programmes.

A last important contextual factor is the ELSA researchers’ home communities. Many of the ELSA researchers are based in disciplinary institutes, such as philosophy, sociology, law and anthropology and these will be important for a number of individual ELSA researchers that need to justify spending time on projects that perhaps cannot be published in the disciplinary communities’ top rated journals. This implies a pressure on these researchers to retain control over the research design in ELSA projects, in order for such projects to qualify also as good disciplinary research.

In short, in order to succeed as a persistent network the ELSA researchers need the support of at least the Research Council and potential project partners from the science and innovation communities, in addition to their home communities. Although the home communities arguably are important I will in the following focus on the Research Council of Norway and the science and innovation communities as this came out as most important in the dialogue conference. As mentioned above, support from such actors can be achieved by selecting, conforming to and/or manipulating the external environments.

### Selecting the right environments

Selecting research *funders* with a favourable outlook on ELSA research is of course important for the ELSA community and the community is already oriented towards the funding sources with explicit openings for ELSA research (see above). Other funding of ELSA projects is rare. Selection of the right *partners* in interdisciplinary projects also seems to be important. In several of the groups stories came up about the cooperation between ELSA researchers and natural scientists and technologists (telling for instance about initial confusion about what the ELSA researchers should be doing in the lab). Different research and innovation environments appears to have different cultures that affect to what extent they view ELSA researchers and the ELSA agendas as legitimate actors and concerns. This perception of legitimacy will affect their willingness to collaborate with ELSA researchers in a way that is meaningful and profitable to the ELSA researchers (in terms of making a difference in the project and producing publishable results).

The question is thus how to select the right actors with which to collaborate. Here the ELSA network may a play a role by inviting potential partners from the natural sciences and innovation environments to events where ELSA researchers present their agendas. From their attendance alone information is yielded about how legitimate the ELSA agenda is perceived. Moreover, feedback forms or other surveys can be used to gain information about what environments express support for the ELSA agenda. ELSA Norway can build on this information to select research and innovation environments that are likely to view the ELSA researchers as legitimate and valuable partners in interdisciplinary projects. A fundamental objection to this selection strategy remains, however, namely that those who are most hostile to ELSA research are those who need it the most. Still, in the perspective of gaining legitimacy by engaging with external actors, choosing those with the least favourable disposition towards ELSA research seems like a hard way to go. Perhaps the hard cases can more easily be won when a certain portfolio of successful projects has been established.

### The conforming strategy

In order to be successful the ELSA network will need to conform to developments in its environment. Indeed, including representatives from the nano and biotech programmes as speakers and participants in the dialogue conference already indicates that such a conforming strategy is sought. Moreover, this was an important reason for asking three of the key note speakers to comment upon responsible research and innovation (RRI). As we have seen RRI is mainly related to European research policy, but is also discussed in the Norwegian BIOTEK2021 and NANO2021 programmes. Integrated projects are perfectly consistent with RRI, but RRI (depending on what definition one chooses of RRI) may have a broader scope than simply integrated research.

A conforming strategy may also involve marketing the ELSA community to the natural scientists and technology developers accepting their growing importance for ELSA research. Making a competence catalogue of ELSA researchers making it easy for natural scientists and technology developers to identify interesting partners was suggested as an activity in the new network. From the group work in the first group session this emerged as an important topic: ‘How to approach the natural scientists, get them on board, get them interested, give them some benefits that they can see? It is important to achieve mutual interest and engagement’. Another group said: ‘It is important to articulate what ELSA researchers can offer when initiatives are launched and cooperation is invited. As a collective, we should show examples of changes that have happened in research, formulate a sort of tool box showing when or where ELSA research should or could make a difference.’ There seemed to be a widespread understanding in the groups that strategies for showing conformation to the environment should be developed and implemented in the network.

In the workshop discussions, however, we found that there was also resistance towards conforming. One of the groups noted that ELSA researchers should remain a critical voice. In the context of RRI it was noted: ‘RRI is itself part of a trend away from regulation, focusing on more voluntary instruments and soft policies, from government to governance. What is the function of RRI as a part of a politically directed trend in society? This topic includes how to study power relations, and the tendency towards increased power of multinational corporations that increasingly work outside national/domestic regulations.’

One should note that RRI in itself is a manipulative strategy because it intends to change the way research is carried out. So when ELSA researchers conform to RRI as an existing contextual structure or trend, they are in fact part of a larger structure used by agents and institutions to manipulate their environment. So RRI is not simply an external structure, but also an instrument for manipulation.

### Manipulating

Manipulating the environment and its expectations may seem like an objectionable strategy, but should rather be understood in a constructive way. A key note speaker at the conference formulated it this way: ‘To create impact you have to act as a change agent’.

In the second group session the participants discussed strategies for the ELSA network. The possibility to organise a conference with NANO2021 and BIOTEK2021 was mentioned. This conference might have a dual strategy; both showing conformity with the expectations of these programmes, but also manipulating their understanding. Offensive strategies for acting as a change agent towards natural science and technology communities should also be considered in the network. This might also include broadening the scope beyond the traditional ELSA technologies. Geo engineering is an emerging ELSA topic in the UK. Also other technology fields may be relevant.

An important discussion in the dialogue conference was on the importance of retaining ‘basic’ ELSA research (stressed for instance by one of the key note speakers), meaning projects defined by ELSA researchers themselves to develop the ethical, legal or social science knowledge bases, in contrast to integrated projects and projects primarily defined by the natural science communities. Allowing such projects to be funded by the innovation programmes would perhaps be the most important goal for strategies for manipulating the environment.

## Building pragmatic, moral and cognitive legitimacy

I have argued that the ELSA community in their attempt to form a stable network need to work to gain legitimacy among the key actors whose support is essential for the survival of the community. In this section I will discuss how the ELSA network needs to work along the three legitimacy dimensions mentioned above; pragmatic, moral and cognitive legitimacy.

### Pragmatic legitimacy

Pragmatic legitimacy will probably be the easiest dimension to consolidate. Pragmatic legitimacy ‘rests on the self-interested calculations of an organization’s most immediate audiences’ (Suchman 1995, p. 578). ELSA researchers have from the Human Genome Project (HGP) in 1989 been in the situation where research funders have perceived a need to accompany research funding to potentially socially controversial technology projects with a smaller sum to ELSA research (or ELSI, as it was called in the HGP). Allocating small sums to ELSA research has been a way for research funders funding new technologies to show social responsibility and get good PR. As such it is in their own interest to fund ELSA research. Critics, such as the report *Taking European Knowledge Society Seriously* (European Commission 2007), claim that taking such an instrumental function compromises the potential critical and constructive function of ethics. Nevertheless, being in such an instrumental position does give pragmatic legitimacy to ELSA research and this may well be strengthened by the current focus on RRI. By requiring RRI approaches in natural science and technology projects funders provide incentives for new cross-disciplinary approaches, increasing the instrumental value of ELSA research. In contrast to RRI, which is still not a very well-known concept among natural scientists and industry, (corporate) social responsibility is a more established concept. There seems to be a good case for demonstrating more clearly towards innovation programmes and scientists that they can gain societal credibility and demonstrate corporate social responsibility by including ELSA research. With offensive communication strategies targeted towards such concepts, ELSA research will have a good chance of gaining pragmatic legitimacy in the eyes of natural science institutes and industry.

Such communication strategies need to include a demonstration of the potential positive impacts of including ELSA researchers in projects. In a key note presentation the importance of documenting impact was stressed: ‘ELSA must have a demonstrable contribution to society and the economy; an effect beyond academia. Demonstrability implies evidence that it has been used in specific audiences.’ In the discussion after the speech, it became clear that having and demonstrating impact was a continuing challenge.

### Moral legitimacy

Suchman says that moral legitimacy ‘reflects a positive normative evaluation of the organization and its activities’ (1995, p. 579). Suchman uses the term ‘moral legitimacy’ to denote legitimacy related to acting appropriately or acting for a greater good. I will make the same distinction as I did in Forsberg 2012b, between moral and normative legitimacy. Suchman’s examples of moral legitimacy lack reference to universal or commonly embraced general societal values. In moral theory, moral judgements need to have a universal character to avoid simply being charged with being a ‘pirate’s creed’ (i.e. a normatively coherent code, but nevertheless not considered moral in a wider, societal context, see Beauchamp and Childress 2012). Rather, the values referred to by Suchman are normative judgements in a particular social context. I will therefore employ the term ‘normative’ instead of ‘moral’ legitimacy.

There are at least two sets of norms that are important for giving the ELSA community normative legitimacy. The first set might well be called moral. ELSA research may easily be seen as acting for the greater good. Especially for the applied ethics part of ELSA research, this is indeed often the researchers’ own intention in the projects.

On the other hand, there is a different set of norms that might retract legitimacy from ELSA research and this is related to its interdisciplinary character. ELSA research is not necessarily interdisciplinary, but, as we have seen, the tendency in the RCN ELSA funding is towards interdisciplinarity and integrated projects. Interdisciplinary research, though becoming ever more important in thematic research programmes, may be perceived to challenge established scientific quality criteria. This holds even more so for transdisciplinary research, that in some interpretations leaves the disciplinary commitments behind altogether and embraces a radical problem based approach where even lay knowledge may be treated as equally important as scientific knowledge (see for instance Häberli et al. 2001, p. 7). If interdisciplinary or transdisciplinary ELSA research is perceived as lacking in scientific quality this might retract from its legitimacy (see e.g. Carew and Wickson 2010 for a discussion of alternative quality criteria for transdisciplinary research). The Research Council, that often requires a problem based approach, is perhaps not likely to have such doubts. However, this might be a challenge both with regard to the potential natural scientist partners and the ELSA researchers’ disciplinary home bases. This is an inherent dilemma in ELSA research.

One of the groups from the first group session stated that ‘the aim should be to preserve the ELSA programme with both basic and integrated projects to maintain autonomy of ELSA researchers’. However, the ELSA community may need to explicitly address the question whether there is a dilemma between quality and problem orientation, keeping in mind that protecting one’s autonomy and integrity does not imply rigidity. The latter point was noted by one of the other groups: ‘Flexibility or framing; there are many examples of ELSA research projects undertaken that are changed underway. Changes should be handled with flexibility and not conceived of as problems. Flexibility increases learning and should be included in the project approach. The Research Council could be involved in facilitating such learning processes.’ Still, the challenge remains for ELSA researchers to clarify the position of ELSA research between disciplinary quality and flexible interdisciplinary problem orientation.

### Cognitive legitimacy

In the literature cognitive legitimacy involves a taken-for-grantedness of the organisation, or at least its comprehensibility, that is, that the audiences understand the place of the organisation and its activities (Suchman 1995, p. 582). This is perhaps the most challenging aspect of legitimacy for the ELSA network and the main reason for this lies in the fact that the ELSA community is in itself broadly interdisciplinary and disagrees on the actual and desired nature of ELSA research. As was pointed out in one of the key note presentations ‘ELSA has suffered from critiques from within instead of increasing the impact of the ELSA field as a whole’. An example mentioned was the Post ELSI Manifesto (Balmer et al. 2012). This understanding was also expressed by one of the groups: ‘the presentation of the field can be difficult to agree on since it is diverse and defined in various ways’.

The dialogue conference was in itself an attempt to establish common understanding across the community, and the experience was that there was a lower degree of conflict in this conference than in earlier ELSA meetings. Still, an attempt was not made to define what ELSA is. In presenting ELSA, one may simply outline the multitude of perspectives and approaches to be found in ELSA research and present this diversity as proof of versatility and flexibility. On the other hand, this minimum solution may not seem to be very effective in helping external actors in getting a clear picture of what ELSA research is, or what they get if they include it in their projects. So it might not increase the cognitive legitimacy of the ELSA community in the eyes of their ‘significant others’. Perhaps it is necessary to engage in the–admittedly difficult–process of coming to a common understanding of ELSA research that is at least quasi-substantial. Options might be to provide a systematic map of the ELSA field topography, or find some common lines of thought that are shared across the field. In an article in the EMBO series Peter Stegmaier (2009) suggests that ‘convergence workers’ like ELSA researchers may even amount to a new profession to serve the needs of modern science governance (p. 115). Such professionalisation seems to require a willingness to define ELSA explicitly.

If it really is impossible to agree on any common profile one may question why there is a need for a network at all. However, there seemed to be a unison enthusiasm for such a network, so some commonalities may be assumed to exist.

In the concluding key note reflections it was noted that the ELSA community should regard itself both as a scholarly community and a community of professionals engaged in common forms of practice. It was suggested that RRI would be more related towards practice; facilitating meeting places for reflection. This is an opportunity for ELSA researchers, but should not be pursued at the expense of the scholarly work. By defining the ELSA network in terms of RRI one may avoid the cognitive problem of disagreements about the identity of ELSA as a scholarly field, but may at the same time reduce the scholarly value of ELSA. Whatever identity is chosen (whether it refers mostly to practice or to a scholarly platform); a process of building a common identity probably requires a strong network coordinator who is willing to drive such a process forward, with all the resistance this is bound to raise.

## Some final reflections on the network and legitimacy

The above discussion of legitimacy has allowed me to answer my initial research question in depth. I have argued that for the ELSA Norway network to be successful it needs to be regarded as legitimate in the eyes of the actors on which ELSA research depend; most importantly the Research Council of Norway, natural science and technology communities and the disciplinary home communities. I have indicated several strategies to gain legitimacy; identifying and selecting potentially supportive environments, and conforming to and manipulating these environments. The ELSA network needs to demonstrate that it can provide more or less tangible benefits, show that it operates according to acceptable principles and spell out its main concepts in a way that is understandable to outsiders.

I believe that laying the ground for cognitive legitimacy is the largest challenge because of the diversity of the field. If cognitive and/or moral legitimacy of the ELSA network is lacking then the preconditions for offensive strategies (for acting as a change agent) are weak. Demonstrating to industry, research funders and the rest of society that ELSA research makes technological innovation better would be the best pragmatic legitimation of the ELSA network one could wish for, and would likely also improve its moral and cognitive legitimacy. If the legitimacy of ELSA research primarily is related to the reputation gains that external agents can harvest, then the whole ethos for ELSA research is emptied and it is hard to argue for the justification of its continued existence. This does not apply to its individual disciplines, but only to ELSA as an interdisciplinary concept.

This leads to the question about what the consequences are if the ELSA network fails to establish itself as an institution with external legitimacy. One may only speculate, but a realistic scenario would be that some stronger local communities (centres and institutes) would continue to carry out ELSA research, and that a number of smaller communities and individual researchers would need to reorient themselves into closely bordering research fields. This would in itself not be a dramatic consequence. The surviving communities might continue to have an interdisciplinary ELSA profile or they would be more specialised into specific ethical, legal or social aspects (avoiding the double interdisciplinarity of ELSA in natural science projects). In the latter case, one may argue that some integrated perspectives might get lost. On the other hand, these more specialised groups may more easily communicate their identity and the potential impacts they may have, and may therefore in fact have more influence in research projects with the natural sciences and industry.

What would be the benefit of keeping together as a wider ELSA community? Apart from being able to work in a concerted way to gain legitimacy along the lines outlined above a few other benefits could be mentioned. In a European (or global) perspective Norway can position itself as a country with substantial and wide-ranging ELSA expertise. This might make international research and industry organisations look to Norway for partnership in European projects. If such a gain is to be harvested this would seem to require active efforts of ELSA Norway, for instance in the form of organising a European conference or actively marketing Norwegian expertise to a wider audience through targeting European researchers and stakeholders through the network website. Indeed, there was already consensus in the conference that this website needs to be in English. Another potential benefit of trying to maintain a broad network is to facilitate recruitment. A larger network may involve a larger pool of resources for project recruitment, and it may also attract younger researchers (a point made at the end of the conference). If this potential gain is to be realised ELSA Norway should prioritise funding ph.d. courses, and perhaps also facilitate researcher mobility between participating institutions. Steady recruitment to the community is important for maintaining and strengthening capacity, quality and diversity.

However, these actions should not overshadow legitimacy building activities towards the key environmental actors. For without the support of funding institutions and potential partners there will not be any continued existence of ELSA research. A potential benefit of a network is that a representative of the whole ELSA community will be a stronger partner in dialogues with the Research Council than single individuals or representatives from some of the centres for ELSA research, and that this may increase the chance of gaining research funding on ELSA premises. However, taking into account the importance of Horizon 2020 one may even argue that ELSA researchers should not only work to establish a national network, but rather consolidate at a European level. The Norwegian ELSA network may be a driver for such initiatives.

Will the ELSA network stand a chance to survive after its two years? This depends on a number of factors; some within and some outside the network’s control. It will be up to the network steering committee to analyse the situation and prioritise network actions in order to gain legitimacy and maximise the chances of survival. The aim of this article has been to contribute with relevant perspectives for achieving this goal.

## Endnotes

^a^When using the term ‘community’ I here mean simply those researchers who have been engaged in ELSA related research, this author included.

^b^http://www.nature.com/embor/focus/convergence_research/index.html.

^c^Referred to as closely bordering because they include some ELSA research (2 to 5% of total funding) as part of the overall focus on natural science and technology projects.

^d^Numbers gathered from their respective webpages on http://www.forskningsradet.no.
